# β-Cell Replacement Strategies: The Increasing Need for a “β-Cell Dogma”

**DOI:** 10.3389/fgene.2017.00075

**Published:** 2017-06-06

**Authors:** Andhira Vieira, Noémie Druelle, Fabio Avolio, Tiziana Napolitano, Sergi Navarro-Sanz, Serena Silvano, Patrick Collombat

**Affiliations:** Centre National de la Recherche Scientifique, Institut National de la Santé et de la Recherche Médicale, iBV, Université Côte d'AzurNice, France

**Keywords:** β-cells, differentiation, stem cells, type 1 diabetes, β-cell markers

## Abstract

Type 1 diabetes is an auto-immune disease resulting in the loss of pancreatic β-cells and, consequently, in chronic hyperglycemia. Insulin supplementation allows diabetic patients to control their glycaemia quite efficiently, but treated patients still display an overall shortened life expectancy and an altered quality of life as compared to their healthy counterparts. In this context and due to the ever increasing number of diabetics, establishing alternative therapies has become a crucial research goal. Most current efforts therefore aim at generating fully functional insulin-secreting β-like cells using multiple approaches. In this review, we screened the literature published since 2011 and inventoried the selected markers used to characterize insulin-secreting cells generated by *in vitro* differentiation of stem/precursor cells or by means of *in vivo* transdifferentiation. By listing these features, we noted important discrepancies when comparing the different approaches for the initial characterization of insulin-producing cells as true β-cells. Considering the recent advances achieved in this field of research, the necessity to establish strict guidelines has become a subject of crucial importance, especially should one contemplate the next step, which is the transplantation of *in vitro* or *ex vivo* generated insulin-secreting cells in type 1 diabetic patients.

## Introduction

Diabetes affects 422 million people worldwide and its increasing prevalence is predicted to reach 552 million patients by 2030 (Whiting et al., 2011; Zhou et al., 2016). The most common feature associated with diabetes is also its principal diagnosis: chronic hyperglycemia. Type 2 diabetes results from a combination of insulin resistance in target organs and defective β-cells (Bergman et al., 2002), while type 1 diabetes is due to the autoimmune-mediated loss of the pancreatic insulin-secreting β-cells, leading to insufficient glucose disposal (WHO, 1999). For both pathologies, the loss of insulin activity causes an imbalance in glucose homeostasis, eventually resulting in multiple cardiovascular complications (Hanefeld et al., 1996; Alwan, 2010; Pascolini and Mariotti, 2012). In the case of type 1 diabetes, the hyperglycemia can be efficiently managed by means of insulin supplementation, but patients still display an overall shorter life expectancy and a relatively altered quality of life (Lind et al., 2014; Morgan et al., 2015). In this context, finding an alternative to daily injections of exogenous insulin has become a crucial research goal. Toward this goal, many current efforts focus on β-cell replacement therapies using different strategies, alongside the development of efficient ways to protect such newly generated cells from the autoimmunology inherent to type 1 diabetes (detailed by Desai and Shea, 2016).

During the last decade, impressive progresses have been made toward the generation of functional insulin-secreting β-like cells (Vieira et al., 2016). Most of the strategies employed initially relied on deciphering the molecular mechanisms underlying β-cell (neo) genesis and applying this knowledge to *in vitro* or *in vivo* (trans) differentiation: the purpose being to drive progenitor cells (either stem cells or multipotent cells) or differentiated cells toward a β-cell phenotype. To validate the identity of the resulting “β-like” cells, a number of tests have been employed, ranging from marker gene analyses to functional challenges. However, while browsing the recent literature, we noticed important differences between the features examined by various authors. Importantly, our survey indicates that the number of key features assessed to establish whether neo-generated insulin-producing cells are indeed “true” β-cells has not progressed in the last years. These observations clearly establish the need of an “initial β-cell profiling.”

## Data analysis

### Methodology

Our analyses were focused on the following β-cell features:

- Glucose Stimulated Insulin Secretion (GSIS) was confirmed when the authors reported at least one insulin and/or C-peptide ELISA measurement increasing upon glucose stimulation, or when an improved response for mice subjected to an intraperitoneal or oral glucose tolerance test was observed. Of note, the sole presence of C-Peptide as a sign of GSIS was not considered.
- Gene expression of *bone fide* β-cell markers was validated when RT-PCR, transcriptomics analyses or immunolabeling was used.
- Mice reverting from an established diabetic state (NOD/Akita background, streptozotocin or alloxan treatment) to stable euglycemia due to the presence of neogenerated insulin-producing cells validated the feature “Hyperglycemia Recovery.” This could be achieved either by *in vivo* transdifferentiation or allogenic transplantation of *in vitro* differentiated cells.

Fifty-nine original publications were manually selected following multiple Pubmed searches (https://www.ncbi.nlm.nih.gov/pubmed/) using the keywords “β-cells,” “pancreas,” “differentiation,” “stem-cells and markers” in various combinations, limiting the searched period from January 2011 to March 2017 (list in Table 1).

**Table 1:** References of the publications analyzed in this survey, listing the source cell types employed for insulin- producing cell neogenesis.

**References**	**Cell type**
***IN VITRO*** **DIFFERENTIATION OF STEM CELLS**	
Iskovich et al., 2011	BM-SC
Thatava et al., 2011	iPSC
Talavera-Adame et al., 2011	mESC
Chen et al., 2011	mESC
Criscimanna et al., 2012	f-LSC
Santamaria et al., 2011	hESC
Jeon et al., 2012	iPSC
Lima et al., 2012	mESC
Bose et al., 2012	hESC
Liu and Lee, 2012	hESC
Wei et al., 2013a	hESC
Wei et al., 2013b	hESC
Tsai et al., 2013	BM-SC
Nair et al., 2014	mESC
Lahmy et al., 2014	iPSC
Ebrahimie et al., 2014	mESC
Niknamasl et al., 2014	iPSC
Shahjalal et al., 2014	iPSC
Hua et al., 2014	hESC
Shaer et al., 2014	M-SC
Van Pham et al., 2014	hPSC
Rezania et al., 2014	hESC
Pagliuca et al., 2014	hPSC
Khorsandi et al., 2015	BM-SC
Jian et al., 2015	M-SC
Pezzolla et al., 2015	hESC
Russ et al., 2015	hESC
Cardinale et al., 2015	iPSC
Agulnick et al., 2015	hESC
Bruin et al., 2015	hESC
Abouzaripour et al., 2016	f-LSC
Salguero-Aranda et al., 2016	mESC
Rajaei et al., 2016	hESC
Manzar et al., in press	iPSC
***IN VIVO*** **CONVERSION OF MATURE CELLS**	
Talchai et al., 2012	Intestinal cells
Banga et al., 2012	Sox9^+^ cells
Al-Hasani et al., 2013	Pancreatic alpha-cells
Courtney et al., 2013	Pancreatic alpha-cells
Chera et al., 2014	Pancreatic delta-cells
Smid et al., 2015	Pancreatic cells
Duan et al., 2015	Intestinal cells
Miyazaki et al., 2016	Pancreatic acinar cells
Yang et al., 2017	Liver cells
Ben-Othman et al., 2017	Pancreatic alpha-cells
Li et al., 2017	Pancreatic alpha-cells
***IN VITRO*** **DIFFERENTIATION OF NON-STEM CELLS**	
Shyu et al., 2011	Pancreatic cells
Zou et al., 2011	Amniotic fluid cells
Ravassard et al., 2011	Fetal pancreatic buds
Kim et al., 2012	Fibroblasts
Akinci et al., 2012	Pancreatic exocrine cells
Lima et al., 2012	Pancreatic exocrine cells
Liu et al., 2013	Liver cells
Kim et al., 2013	Pancreatic duct cells
Wilcox et al., 2013	Pancreatic α-cells
Bouchi et al., 2014	Gut progenitor cells
Sangan et al., 2015	Pancreatic α-cells
Corritore et al., 2014	Pancreatic duct cells
Yamada et al., 2015	Pancreatic duct cells
Teichenne et al., 2015	Pancreatic acinar cells
*mESC: mouse embryonic stem cells*	*BM-SC: bone marrow stem cells*
*iPSC: induced pluripotent stem cells*	*BT-SC: biliary tree stem cells*
*hESC: human embryonic stem cells*	*Endo-SC: endometrial stem cells*
*f-LSC: fibroblast-like limbal stem cells*	*EC-SC: embryonal carcinoma stem cells*
*hPSC: human pluripotent stem cells*	*M-SC: mesenchymal stem cells*

### Validation of β-cell features

Aiming to summarize the β-like cell features assessed, a survey of the recent literature reporting β-like cell neogenesis was conducted by analyzing all the data provided by the authors in order to deliver an accurate compilation. In the resulting 59 original publications, all the properties used to characterize neo-generated β-like cells were inventoried, ranking them by year of publication and the frequency of their use as a validation tool (Table 2).

**Table 2:** Summary of the features assessed in neo-generated β-like cells ranked both chronologically and by frequency.

**Year**	**Insulin (%)**	**Pdx1 (%)**	**GSIS (%)**	**Nkx6.1/6.2 (%)**	**Glut2 (%)**	**NeuroD1 (%)**	**MafA (%)**	**Pax4 (%)**	**HG recovery (%)**	**Pax6 (%)**	**Glucokinase (%)**	**Foxa2 (%)**	**Isl1 (%)**	**PC1/3 (%)**	**Nkx2.2 (%)**	**Kir6.1/6.2 (%)**	**Sur1 (%)**	**PC2 (%)**	**Hlxb9 (%)**	**Iapp (%)**	**Urocortin3 (%)**
2011	100	100	75	50	75	63	38	13	50	63	38	25	25	13	13	13	25	0	13	0	0
2012	100	90	80	60	70	80	70	80	50	40	40	60	30	30	30	30	30	30	20	20	0
2013	100	100	88	50	75	63	75	63	63	63	13	13	38	38	13	13	0	13	13	0	0
2014	100	100	92	85	38	38	46	38	31	31	38	15	46	31	23	23	23	23	23	15	23
2015	100	83	92	42	42	33	50	50	42	17	42	25	17	33	33	25	25	17	0	25	0
2016	100	100	100	50	25	0	0	0	25	0	25	75	0	0	50	25	0	0	0	0	0
2017	100	75	100	75	25	50	25	50	75	25	0	0	25	25	0	25	25	0	0	0	25
Total	100	93	88	59	53	49	49	46	46	36	32	29	29	27	24	22	20	15	12	12	7

*For each year, the percentage of publications having validated a particular feature is displayed. GSIS, Glucose Stimulated Insulin Secretion; HG recovery, HyperGlycemia recovery, see Methodology for a description of the validation criteria*.

*Color gradient reflecting the percentage of validated features*.

#### Insulin and β-cell function

Expectedly, insulin expression was the only feature commonly displayed by all reported neo-generated β-like cells. Interestingly, the responsiveness of such β-like cells to glucose stimulation was assessed in 88% of the publications analyzed, indicating a satisfying physiological response for most of these newly generated cells. However, the recovery upon induced hyperglycemia was validated in only 46% of the publications listed. In the case of insulin-secreting cells generated *in vitro* and challenged *in vivo*, this can most likely be attributed to transplantation-related issues and the need to host immune-deficient mice, *in vitro* differentiated allogeneic or xenogeneic cells being rejected upon graft in wild-type animals. In the case of *in vivo* transdifferentiation, on the contrary, the immunological rejection is bypassed by the creation of autologous β-like cells, and consequently the hyperglycemic recovery was assessed in all publications except one.

#### Transcription factors

The *Pdx1* gene appeared second in ranking, while being a disputed proof of completed β-cell differentiation (Table 2). Indeed, during the course of pancreas morphogenesis, *Pdx1* is first detected in all pancreatic progenitor cells, its expression being subsequently detected in mature β-cells (Ahlgren et al., 1996, 1998). *Pdx1* should therefore not be considered as a mature β-cell marker in approaches aiming at recapitulating pancreas development, as one cannot exclude that an undifferentiated proportion of the cells still expresses this transcription factor. We consequently suggest that its presence should solely be assessed in insulin-secreting cells using double labeling.

During pancreas development, an initial expression in pancreatic/endocrine precursors and a subsequent expression in mature β-cells is in fact a feature displayed by numerous transcription factors considered as *bona fide* β-cell markers. Indeed, HlxB9, Nkx6.1, Pax4, MafA, Nkx2.2, Isl1, NeuroD1, Pax6, Foxa2 are all involved in pancreas organogenesis, their expression being maintained at adult age in β-cells (for the first four) and additional cell subtypes (for the remaining-Ahlgren et al., 1997; Naya et al., 1997; Sander et al., 1997, 2000; Sosa-Pineda et al., 1997; Sussel et al., 1998; Li et al., 1999; Edlund, 2002; Henseleit et al., 2005; Zhao et al., 2005). It is thus necessary to validate their expression in insulin-secreting cells either using (q)RT-PCR after FACS sorting or immunohistochemical analyses coupled to insulin detection.

#### Enzymes and hormones

In the pancreas, glucokinase is expressed in mature α- and β-cells, such enzyme being involved in glucose-sensing (Pierreux et al., 2006). PC1/3 and PC2 correspond to enzymes essential for proinsulin processing and are thus necessary for the normal function of mature β-cells (Marzban et al., 2004; Ugleholdt et al., 2006). Iapp is co-released with insulin by β-cells and acts as a satiation signal (Akesson et al., 2003), while urocortin3 is a hormone secreted by β-cells, acting to induce somatostatin secretion by δ-cells (van der Meulen et al., 2015). Altogether, these proteins are involved in the β-cell metabolism and function, and therefore should mostly be expressed only in mature insulin-secreting cells. Accordingly, they represent markers of the maturation state of differentiated insulin-secreting cells and they should therefore be tested in a more systematic way to ascertain a terminally-differentiated β-like cell phenotype.

#### Channels

Even though they are not markers of differentiation or maturation per se, potassium channels Kir6.1 and Kir6.2 and ATPbinding cassette channel Sur1 are required for proper insulin secretion (Proks et al., 2002; Kefaloyianni et al., 2013). Coupled to GSIS assessment, the presence of these proteins should be used in order to establish optimal β-like cells response to glucose.

## Discussion

Following the outstanding progresses made in the fields of stem-cell differentiation and *in vivo* trans-differentiation, human applications appear increasingly conceivable. However, one could only contemplate such an exciting clinical outcome after ensuring that the neo-generated insulin-secreting cells are genuine and could therefore fully replace endogenous β-cells. The features displayed in Table 2 rank the common features classically assessed in neo-generated insulin-secreting cells which, taken together, could theoretically constitute an “initial profiling” for β-like cells. Obviously, numerous additional aspects of the β-cell phenotype should be considered when aiming at establishing a standard validation protocol for β-like cells.

Regarding the prerequisites listed in Table 2, as previously discussed, appropriate levels and correct localization of the β-cell-specific marker genes undoubtedly should be confirmed by immunohistochemistry using double labeling, especially in the case of developmental transcription factors. Concerning insulin itself, since its release in response to a stimulus is the main property requested from β-like cells, careful examination of its glucose-stimulated secretion and proper storage of insulin are essential. For the latter, the visualization of secretory vesicles by electronic microscopy appears as a valuable tool. Combined with the PC1/3, PC2, and C-peptide expression analyses, proper processing of proinsulin could be convincingly demonstrated. In addition, the analysis of single insulin-producing cells could provide cues on their ability to behave as endogenous β-cells.

While global proteomic and transcriptomic analysis of neo-generated cells would give detailed information about their state of differentiation, one of the main issues is the heterogeneity of β-cells both in human and rodents (Rutter et al., 2015; Dorrell et al., 2016; Roscioni et al., 2016). A detailed analysis of these aspects, as well as a list of putative routine experiences, are described by James D. Johnson in his elegant review detailing the remaining steps prior to reaching clinical applications (Johnson, 2016). This report provides a thorough analysis of the current state-of-the-art from the point of view of a β-cell biologist, also highlighting the need for standardized protocols validating β-like cells functionality.

In addition to the initial profiling of neo-generated β-like cells, systematic single-cell next generation transcript sequencing (RNA-seq) would be the most decisive validation for β-like cells, providing the complete expression profile of these cells and thus their state of differentiation. Importantly, this transcriptomic phenotyping would not only assess the activation of necessary features, it would ensure the correct repression of non-β-cell genes, including the disallowed genes known to interfere with appropriate β-cell functionality (Pullen and Rutter, 2013; Lemaire et al., 2016; Pullen et al., 2017).

**Figure 1:**
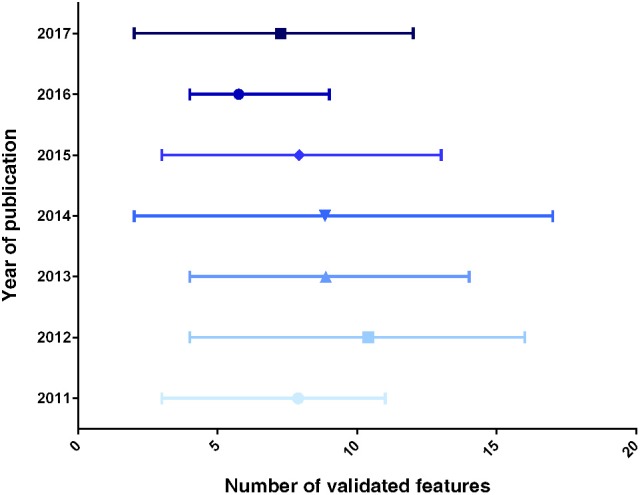
Graphical representation of the number of β-like features validated, per year of publication. The data displayed represent the average number of validated markers ± range, for each year of publication (from the list displayed in Table 2).

A chronological display of the average number of features assessed in neo-generated β-like cells is provided in Figure 1. Importantly, this ranking clearly shows a scattering in the number of validated features, the average values not increasing in time (which would be indicative of an enhanced scrutiny cells over time). These discrepancies clearly reflect the lack of a canonical list of features to be validated. We thus propose to systematically assess most (if not all) of the features displayed in Table 2 as an initial roadmap toward the establishment of the β-cell identity.

## Author contributions

AV conceptualized the study, chose the methodology and wrote the original draft. ND, FA, TN, SN, and SS contributed to the formal analysis and investigation. PC validated the results, supervised the study, edited the draft and reviewed the final version prior to submission.

### Conflict of interest statement

The authors declare that the research was conducted in the absence of any commercial or financial relationships that could be construed as a potential conflict of interest.
